# Ethical considerations in the use of social robots for supporting mental health and wellbeing in older adults in long-term care

**DOI:** 10.3389/frobt.2025.1560214

**Published:** 2025-03-31

**Authors:** Lillian Hung, Yong Zhao, Hadil Alfares, Parsa Shafiekhani

**Affiliations:** ^1^ School of Nursing, University of British Columbia, Vancouver, BC, Canada; ^2^ IDEA Lab, University of British Columbia, Vancouver, BC, Canada

**Keywords:** ethical challenges, social robots, long-term care, dementia, older adults

## Abstract

Social robots are increasingly being utilized to address mental health challenges in older adults, such as depression, anxiety, and loneliness. However, ethical concerns surrounding their use are insufficiently explored in empirical research. This paper examines the ethical challenges and mitigation strategies for implementing social robots in long-term care settings. Drawing from insights gained from research across two Canadian studies involving Paro and Lovot, we highlight the critical role of an equity-focused approach to ensure the ethical use of social robots. We advocate for the respectful inclusion of the voices and desires of marginalized groups, such as older adults with dementia. Key ethical issues discussed include inequitable access, consent, substitution of human care, and concerns about infantilization. Our empirical work offers practical strategies to navigate these challenges, aiming to ensure that social robots promote mental health and wellbeing in an ethically responsible manner for older adults living in long-term care.

## 1 Introduction

**FIGURE 1:**
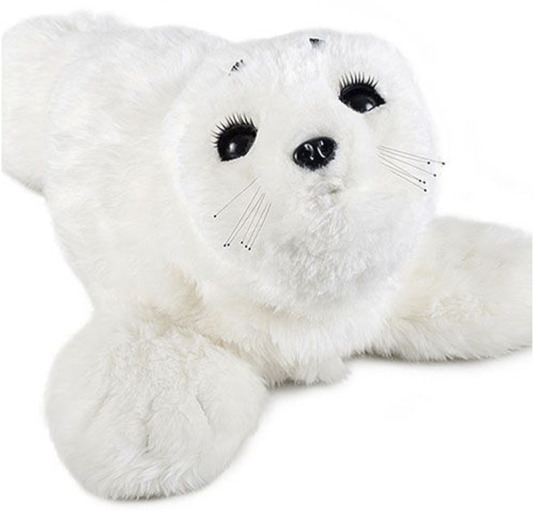
Paro.

Social robots, designed to engage with older adults through interactive and emotional responses, have been reported as a promising tool in long-term care (LTC) (Hung et al., 2021; Yu et al., 2022). Scandinavian countries have been leaders in adopting social robotic technology in LTC to support the mental health and wellbeing of older adults. Countries like Denmark, Sweden, and Norway have integrated social robots into their healthcare and eldercare systems (Blindheim et al., 2023; Dinesen et al., 2022; Persson et al., 2024). The primary goal is to support older adults’ mental health and wellbeing (Grunfelder et al., 2020). Similarly, social robots like Paro and Lovot have been used in Canadian LTC to support older adults’ mental health and wellbeing (Hung et al., 2019a). They are designed to provide companionship and emotional support and enhance overall wellbeing, particularly for those who may experience social isolation or mental health challenges. Paro, developed by Japan’s AIST, is an advanced therapeutic robot designed to resemble a baby seal (Figure 1), providing the benefits of animal therapy in environments where live animals are impractical, such as hospitals and LTCs. Paro has demonstrated positive benefits of reducing stress and enhancing socialization among residents and caregivers, improving overall wellbeing (Takayanagi et al., 2014). Equipped with tactile, light, auditory, temperature, and posture sensors, Paro can detect and adapt to user interactions and learning behaviors that encourage positive engagement, which makes it a valuable tool in dementia care (Hung et al., 2019b). Paro has been shown to reduce behavioural and psychological symptoms of dementia, perception of pain, anxiety, and depressive symptoms, as well as reduce medication use (Rashid et al., 2023).

**FIGURE 2:**
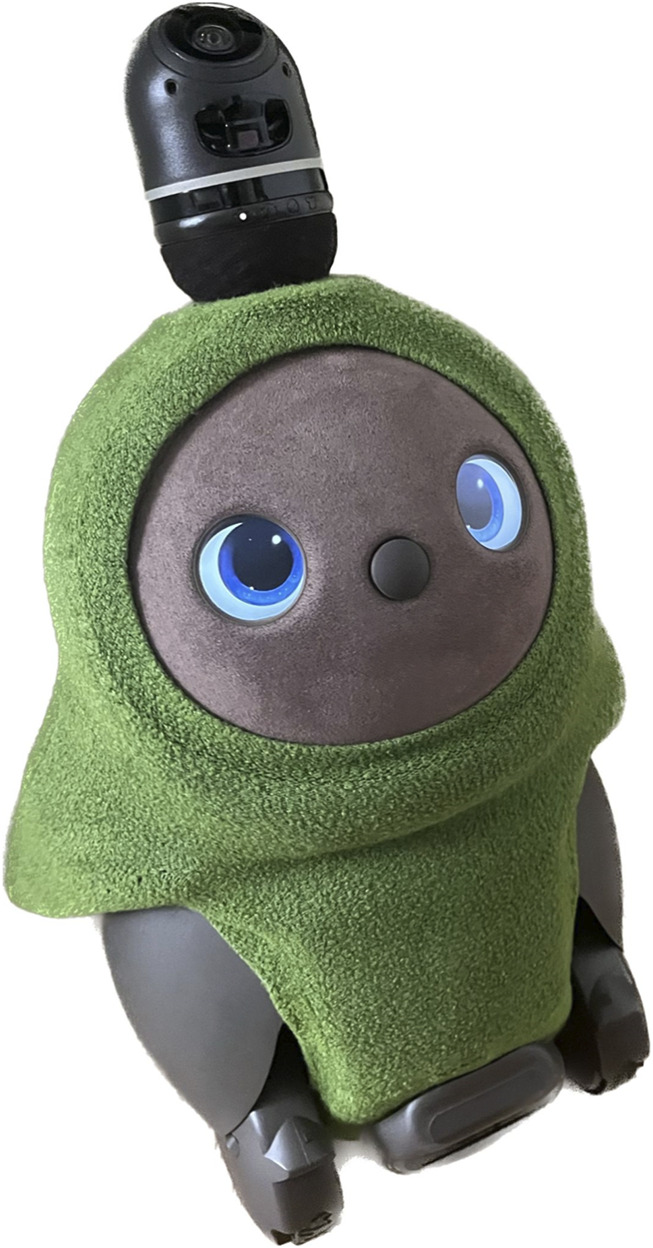
Lovot.

Lovots (Figure 2) are designed with three key features that make them emotionally engaging companions. First, their emotional interaction capabilities are powered by sensors and artificial intelligence (AI), allowing them to recognize faces, follow movements, and respond to touch, creating lifelike connections. Second, their mobility enables them to move on wheels, follow users, and explore their surroundings. They can recognize their owner, respond to their name, and react to sounds using cameras and microphones. Lastly, Lovots’ affectionate design allows them to respond positively to gentle petting and hugs, enhancing the sense of physical and emotional bonding (GROOVE X, 2024). Lovots can move toward residents, seeking physical interaction and responding to affection, which helps promote engaging social interactions (Mononen, 2019). In Singapore, Tan et al. (2024) found that Lovot provided valuable psychosocial support to single older adults by fostering emotional bonds and companionship. In Denmark, Dinesen et al. (2022) showed that residents living with dementia responded positively to Lovot, and healthcare professionals viewed the robots as a useful communication tool.

Although social robots offer a myriad of benefits, caregivers and residents have voiced their concerns with their implementation. A previous study by Wong et al. (2024) identified barriers to adopting AI-enabled robots in LTC, including ethical considerations. Exploring the ethical challenges of social robots in LTCs is crucial because these technologies directly interact with older adults, who may experience cognitive impairment, loneliness, and physical dependency. For instance, older adults with dementia may need to be reminded that the robot is a machine controlled by algorithms rather than a real animal pet. Boada et al. (2021) reported ethical issues of using social robots, including deception, consent, autonomy, access, and emotional dependency. Researchers should move beyond the conventional procedures of Institutional ethics (University Research Ethics Board), which tend to be limited to informed consent, capacity, and protection. Institutional ethics do not fully capture the complexities of fostering equitable and meaningful participation among LTC residents throughout the research process. Here, we argue that everyday relational ethics offers a practical framework emphasizing ongoing, dynamic engagement with participants, ensuring their voices and perspectives are heard and valued throughout the research process (Mikesell, 2023). This approach is especially critical in LTC settings, where cognitive and physical challenges can make it difficult for residents to research and experience social robots’ benefits. This paper addresses this gap by highlighting the ethical challenges and practical strategies, drawing on our two empirical studies using social robots, Paro and Lovot, in Canadian LTC homes. Table 1 shows our reflection on these two concepts.

**TABLE 1:** Reflection of empirical studies on Lovot and Paro.

Institutional ethics	Everyday relational ethics
Purpose Institutional ethics focus primarily on protecting vulnerable populations and ensuring that participants are not harmed. This framework is designed to uphold standardized ethical principles, especially in research settings where participants may be at risk	Purpose Everyday relational ethics take a more holistic and relational approach, emphasizing the importance of considering the broader context in the care setting, focusing on older adults’ wellbeing beyond avoiding harm. This approach attests to the participant’s daily life and social relations
Consent In institutional ethics, consent is typically obtained at the beginning of the research process and is viewed as a formal, often one-time event. It often requires participants to be capable of signing consent forms, usually in English, which may exclude non-English speakers or individuals who struggle with formal documentation	Consent Consent in everyday relational ethics is ongoing, recognizing that participants’ ability to provide consent may change over time. Researchers must pay attention to non-verbal cues for assent or dissent, which is particularly important when working with older adults who may have cognitive impairments or communication difficulties
Researcher-Centered Institutional ethics prioritize traditional research protocols, requiring participants to follow predetermined procedures with inclusion and exclusion criteria. The power dynamic is researcher-driven; participant compliance is necessary to protect study fidelity	Participant-Centered Everyday relational ethics place the participant at the center of the research process, focusing on meeting their emotional and physical needs. Researchers must accommodate participants’ evolving needs and preferences, respecting their experiences and rights for equity

Everyday relational ethics emphasizes the importance of relationships and context in making ethical decisions (Mann and Hung, 2019). It focuses on the dynamics between individuals, their environment, and the broader social structures that shape their experiences. In everyday relational ethics, care, empathy, and mutual respect are central, recognizing that ethical decisions are not made in isolation but are deeply influenced by the interactions and relationships between people (Banks et al., 2013). Equity, a relevant concept to everyday relational ethics, refers to fairness and justice in ensuring that individuals or groups have access to the resources, opportunities, and support they need to thrive, regardless of their starting point or disadvantages (Smith and Stajduhar, 2024). To advance equity, more must be done to support disadvantaged and vulnerable groups, such as individuals with advanced dementia. Unlike equality, which suggests treating everyone the same, equity acknowledges that people have different circumstances and requires the allocation of resources and support tailored to these differences to achieve fair outcomes. In this context, we emphasize the critical importance of prioritizing equity in the ethical adoption of robotics in LTC. This approach ensures that all residents, including those with cognitive impairments and language barriers, are afforded opportunities to benefit from technology. An inclusive system empowers every individual, regardless of their disabilities, to experience the full advantages of innovation in care. This paper explores ethical challenges experienced during the implementation of social robots Paro and Lovot for the mental health and wellbeing of residents living in Canadian LTC. Implications are discussed to inform future research and practice to promote the mental health and wellbeing of LTC residents in social robot research.

## 2 Methods

Our research team has studied Paro in four Canadian LTC homes since 2016 (Hung et al., 2021). Furthermore, we investigated the use of Lovot for a year (2023-24) (Hung et al., 2025). Both studies received ethics approvals from the local university (H23-01683 and H18-03483). Our research team, consisting of researchers and trainees, collaborated with older adult partners to support local LTC homes in using robots to improve residents’ mental health and wellbeing. This implementation followed a Collaborative Action Research methodology involving older adults, families, staff, and operational leaders to co-develop technology adoption strategies. The robot sessions varied from 5 to 30 min, in individual and group formats, with support from families, students, recreational, rehabilitative, and nursing staff.

Large and urban care homes included one privately funded and three publicly funded institutions. Most residents in these homes are elderly, with multiple co-morbidities and disabilities, including dementia. Nurses, care workers, and dietary and recreational personnel provide various services, including medical care, personal support, and recreational activities. This social robot research program aimed to create meaningful, enjoyable experiences to promote social engagement and support older adults’ mental health and wellbeing.

In the Lovot study, 36 participants were recruited. The majority of participants were between 80 and 90 years old (86%), with a small proportion aged 91–100 (6%) and older than 100 (8%). The average age of participants was approximately 87 years. Most participants were Caucasian (94%), and 58% were female. In terms of mobility, 61% used a wheelchair, 27% used a walker, 8% were independent, and 3% were bedridden.

In the Paro study, ten participants ranged from 60 to over 85 years old, with the largest group aged 76–85 (60%). The average age was approximately 78 years. Sixty percent were male, and 40% were female. In terms of health conditions, all participants had dementia, with 20% in the early stage, 50% in the middle stage, and 30% in the late stage. The majority were Caucasian (70%), with 20% South Asian and 10% Black participants.

In our research, both Paro and Lovots have shown positive effects in reducing feelings of loneliness and improving mental health by offering emotional companionship and promoting positive interactions. For example, Paro’s calming influence has been shown to reduce symptoms of stress and anxiety in older adults with dementia (Hung and Chaudhury, 2019). At the same time, Lovot encourages emotional bonding, which can help bring joy and positivity to older adults (Hung et al., 2023). In addition to emotional support, these robots promote cognitive engagement. Interacting with robots like Paro and Lovot stimulates conversation, memory recall, and attention, contributing to cognitive health. Their ability to respond to touch and voice helps maintain mental stimulation, creating moments of joy and connection that enhance overall wellbeing.

## 3 Results

Our empirical studies indicate four key ethical challenges associated with implementing social robots in LTC facilities: inequitable access, consent, substitution of human care, and concerns about infantilization. For each ethical challenge, a mitigation strategy has also been suggested to address ethical support for older adults in LTC settings.

### 3.1 Inequitable access

#### 3.1.1 Challenge

Inequitable access to social robots is a common issue in LTC settings, and language barriers often exacerbate these inequalities. Paro is introduced to residents in many facilities by recreation staff, who typically speak English. As a result, only residents who can communicate in English are often invited to participate in sessions with Paro. Older adults who do not speak English are less likely to have the opportunity to interact with the robot, leading to exclusion and a missed opportunity for emotional support and companionship. This disparity disproportionately affects residents from immigrant or minority backgrounds, further marginalizing those who may already experience limited social engagement. For example, Paro was introduced as part of a recreational therapy program in one LTC home. The staff, while well-intentioned, would invite only English-speaking residents to participate, as they felt more comfortable facilitating conversations and interactions in English. Mrs. Zhang, an 85-year-old resident who speaks Mandarin, often sat by herself in the common area, observing the Paro sessions. She smiled when she saw others interacting with the robot. The staff, unaware of her feelings and potential interest, assumed that because they could not communicate easily in English, she would not benefit from the robot. The first author researcher invited Mrs. Zhang to pet Paro; Mrs Zhang hugged and kissed the robot. She expressed joy and excitement and told Paro about her white cat and how much she missed her family. This story highlights how language barriers can lead to the exclusion of non-English-speaking groups in LTC, limiting their access to technology’s potential emotional and social benefits.

#### 3.1.2 Mitigation strategy

Everyday relational ethics emphasizes addressing such inequities by advocating for inclusive practices that consider language and cultural differences. To ensure that non-English speaking residents like Mrs. Zhang can also benefit from social robots, involving staff who speak different languages or providing interpretation services is crucial. Inequitable access to the robot can arise from staff assumptions and decision-making about who should engage with the robot. Staff may assume that those residents who do not speak English are less capable of engaging or more cognitively impaired. This implicit bias led to exclusionary practices, limiting access for non-English-speaking residents even though Paro’s interaction is largely non-verbal and does not require spoken language comprehension. This highlights an equity issue in implementation, where access is determined not by the technology itself but by staff perceptions and practices. Addressing this requires staff training and awareness to ensure all residents have an opportunity to engage with Paro. Staff should be trained to use non-verbal cues and relational engagement to involve all residents in the program. This strategy not only increases access but also ensures that the voices and needs of disadvantaged groups are privileged, allowing all residents to participate meaningfully in social robot programs.

### 3.2 Consent

#### 3.2.1 Challenge

In traditional institutional ethics, the need to protect older adults—particularly those with cognitive impairments—can conflict with supporting their autonomy and dignity. For example, Mr. Lee, an older LTC resident with advanced dementia, cannot sign an informed consent form and has no family or close contacts to act as a proxy. This resident is already at risk of isolation due to cognitive decline and the absence of a support network. By excluding Mr. Lee, researchers can perpetuate inequities in care and technology adoption. The Lovot robot has shown benefits in improving wellbeing, reducing loneliness, and enhancing emotional support for residents in the same LTC home. However, when individuals with cognitive impairments and without familial support are systematically excluded from research, they are denied the opportunity to benefit from these advancements. This exclusion not only undermines the principles of equity and justice but also diminishes the inclusivity and generalizability of research findings, making it difficult to understand how social robots might be adapted to meet the diverse needs of all residents. The exclusion criteria in institutional ethics and practice disproportionately affect disadvantaged groups, particularly individuals with dementia, who are denied access to beneficial technologies based on their inability to sign informed consent.

#### 3.2.2 Mitigation strategy

Researchers should consider developing ethically robust strategies for inclusive participation while respecting the rights and dignity of those who cannot provide traditional forms of consent. For residents without family support, this could include employing a relational process consent approach where consent is revisited throughout the research, observing non-verbal cues to gauge comfort and engagement, and involving care staff who are familiar with the resident’s preferences and behaviours. For instance, a care aide who worked closely with the resident, like Mr Lee, observed his reactions to group activities and interactions with others. Although the resident cannot verbally express interest or sign a consent form, the staff member noticed that the resident consistently smiled and leaned forward when observing others interacting with the robot. The staff member also recalled how the resident enjoyed tactile stimuli and responded positively to the robot with engaging movement—a key feature of the Lovot robot. Recognizing these cues, the staff member advocated for the resident’s participation in the study. During the sessions, the researcher and staff monitored the resident’s reactions, looking for signs of discomfort or disinterest, such as withdrawing or turning away, while noting positive responses like reaching out to touch the robot or laughing joyfully. This practice ensured that the resident’s non-verbal desire to engage was respected. By involving staff who know residents well in interpreting non-verbal cues and advocating for their participation, researchers can create more inclusive and equitable studies. This approach acknowledges the unique needs of residents with advanced dementia or communication barriers and ensures they are not excluded from opportunities to benefit from emerging technologies. It also highlights the importance of relational care and trust in creating ethically sound research practices that prioritize equity and inclusivity. By prioritizing their voices and tailoring the consent process to include these vulnerable groups, we protect their autonomy and promote their right to participate in research and care decisions. This approach upholds the relational aspects of care and values the person’s ongoing participation in meaningful ways.

### 3.3 Substitution of human care

#### 3.3.1 Challenge

Concerns about overreliance on robots in caregiving have sparked debates about whether technology might replace the essential human touch in LTC settings. While social robots like Paro and Lovot have proven effective in promoting social engagement and fostering relationships, there is a risk that they could be used as substitutes for human interaction, particularly in under-resourced or understaffed aged care facilities. This concern is especially pressing for residents who already face isolation due to cognitive impairments, disabilities, or a lack of family support. Everyday relational ethics remind us that caregiving is inherently relational, and no robot can fully replace the depth of human connection. For residents with dementia or other cognitive impairments who may struggle to form connections or communicate, the consistent presence of empathetic human caregivers is vital for their wellbeing. The overreliance on robots could lead to the dehumanization of care, overlooking the unique psychosocial needs of LTC residents and exacerbating existing inequities, as residents who are less able to advocate for themselves may disproportionately experience a reduction in human interaction. During our research, team members and staff participated simultaneously, and no issues related to the substitution of human care were observed. However, after the project concluded, when we left the robot at the LTC home and later retrieved Paro, we found that it had not been kept by staff but was instead placed directly with residents without supervision. While there were no safety concerns with Paro, the absence of structured oversight suggests a potential risk of social robots being used as a passive substitute for human interaction rather than as a complementary tool in care settings.

#### 3.3.2 Mitigation strategy

Robots should never be seen as replacements for human care but rather as tools to complement and enhance human interaction, particularly in ways that protect and prioritize equity for socially disadvantaged or cognitively impaired individuals. Their integration into care practices must center on the unique needs, desires, and rights of each individual, ensuring that those who are most vulnerable are not marginalized or further disenfranchised. For example, in both our Paro and Lovot projects, staff and researchers were always present as facilitators during interactions with the robots. Their role was to guide and support residents, ensuring that the robots were used in ways that encouraged engagement and promoted individual agency. These facilitators helped bridge any gaps in understanding or interaction, ensuring that the technology complemented human care rather than substituted for it. By employing robots to support—rather than replace—meaningful human interactions, we uphold the principles of equity and person-centered care, preserving the vital relationships between residents and caregivers as central to their wellbeing. Robots can contribute to fostering connection, but only when they are used ethically, relationally, and in a manner that amplifies the voices, agency, and dignity of all individuals, particularly those from disadvantaged groups.

### 3.4 Concerns about infantilization

#### 3.4.1 Challenge

The design of social robots, particularly those with childlike or animal-like characteristics, may make some residents feel infantilized (Fardeau et al., 2023). This can be especially problematic for people with dementia, who may perceive robots as demeaning or inappropriate for their age and condition. Everyday relational ethics calls for a deeper understanding of residents’ personal contexts, desires, and self-perceptions, ensuring that their dignity is preserved in the design and implementation of care technologies. In our study, most residents did not report feelings of infantilization; on the contrary, Lovot and Paro were highly welcomed in LTC homes. However, concerns were raised by two female residents in the Lovot study and one male relative in the Paro study. These instances were documented through direct interviews and observations. In the Lovot study, two residents told us that the lovot robots are meant for others—those as “more needy” or less capable. In the Paro study, a resident (mother) was delighted to join a group activity where she could interact and play with the Paro robot, but her son was uncomfortable and perceived the activity as “embarrassing” or “childish.” While the son may have viewed the interaction as inappropriate for her age, the mother found it socially enjoyable and stimulating in the group activity. After a family conversation between the siblings, the son recognized her mother’s agency and respected her desire to participate in the social robot activity, honouring her individuality and preserving her dignity.

#### 3.4.2 Mitigation strategy

Addressing this challenge requires an inclusive approach to robot design and implementation that considers and respects the perspectives of all users, particularly disadvantaged groups like those with dementia. The above example underscores the importance of prioritizing the older adult’s perspective rather than projecting assumptions onto their experiences. Facilitators can play a key role by ensuring the design and use of social robots are framed as tools for engagement, choice, and connection rather than infantilization. By creating opportunities that validate the preferences and autonomy of older adults, care settings can mitigate feelings of stigma and promote a sense of inclusion and respect. Furthermore, encouraging open dialogue with family members and caregivers about the value of these interactions can help reduce misunderstandings and support a more inclusive approach to care.

To ensure that social robots effectively meet the needs of older adults while fostering dignity and engagement, it is essential to actively involve older adults in the product development and implementation process. By seeking their input and incorporating their feedback, we can create robots that align with their preferences and aspirations, reducing the risk of fostering feelings of infantilization or disempowerment. Inclusion in the design process allows older adults to have a voice in shaping technologies that impact their daily lives, ensuring that these tools reflect their unique needs, desires, and self-perceptions. The relational aspect of care demands that we treat each resident as an individual, actively listen to their voices, and consider their diverse contexts. Through this collaborative approach, technology can be developed to empower older adults, enhance their sense of autonomy, and improve acceptance and engagement while also safeguarding their dignity. Involving older adults at every stage of development—from initial design to real-world implementation—not only results in more meaningful and effective tools but also demonstrates respect for their expertise and lived experiences. This participatory approach ensures that social robots become facilitators of connection and empowerment rather than sources of discomfort or exclusion.

## 4 Discussion and implications

Based on our empirical studies, this paper explores key ethical challenges associated with implementing Paro and Lovot robots in LTC facilities. The use of social robots in LTC offers significant potential to support the mental health and wellbeing of older adults, but ethical considerations must guide their implementation to ensure that these technologies promote dignity, autonomy, and equity. This paper bridges a critical gap in the literature regarding how equity should be addressed in their research and implementation. We draw attention to the unique needs and special conditions of LTC residents, such as cognitive impairments, diversity, and a lack of support. Without addressing these factors, the integration of social robots risks marginalizing the individuals they aim to support, exacerbating existing inequities in care. We aim to make a contribution by emphasizing the importance of equity in the use of social robots among LTC residents.

By foregrounding the perspectives of residents, particularly those with cognitive impairments or from underrepresented groups, this work highlights the necessity of participatory approaches in the development of care technologies. The inclusion of residents’ voices ensures that robots are not only functional but also empowering, aligning with the values and preferences of those they are intended to serve. As suggested by Wangmo et al. (2019), both verbal responses and non-verbal cues should be considered when obtaining research consent. Therefore, our work advocates for involving staff who are familiar with the residents and are able to interpret their non-verbal cues to gauge their interest in engaging with social robots. This is significant as implementing a consent process that accounts for residents’ cognitive abilities and communication barriers ensures that vulnerable groups aren’t excluded from benefiting from emerging technologies that have the potential to positively impact their mental health and wellbeing. However, there are limitations associated with obtaining non-verbal consent or assent, including the risk of misinterpretation of non-verbal communication. Therefore, it is important that researchers adhere to best practice guidelines for obtaining assent and respecting dissent, as outlined by Black et al. (2010), Dewing (2007), and Pyer and Ward (2024). These best practice guidelines ensure that people with dementia are respected, given opportunities to participate meaningfully in research, and that their assent is actively sought, and dissent respected. Thoughtful and meaningful safeguards must be in place to protect residents’ rights and autonomy.

Non-verbal cues can be ambiguous. A scoping review found that when verbal communication is not possible, nurses rely on haptics, kinesics, proxemics, and colloquial gestures to interact with residents, but these methods can lead to misinterpretation (Wanko Keutchafo et al., 2020). Addressing these challenges requires a person-centered approach, where effective communication depends on the frontline staff’s familiarity with residents, proper training, and the implementation of strategies that prioritize individualized care (Östlund et al., 2023).

Furthermore, this work, along with the findings of studies such as Chita-Tegmark and Scheutz (2021), contributes to a growing consensus that social robots can enhance human interactions rather than substitute them. Our work builds on this understanding by emphasizing the importance of having social robots as supporters of meaningful connections built between caregivers and residents, which are essential for addressing mental health problems such as loneliness among residents. Our study results highlight the need to address language barriers through interpretation services and consider cultural differences in LTC settings. Through such inclusive practices, we not only ensure that non-English speaking residents get the opportunities to engage with social robots, but we ensure that the unique needs of disadvantaged groups are addressed and effectively met.

It is important to note that while this paper identifies the ethical challenges associated with using Lovot and Paro, other social robots with different features may present other ethical issues in LTC settings (Körtner, 2016). Additionally, cultural factors may impact individuals’ reactions to social robots, resulting in varying challenges from those discussed here. For example, one study found that nurses tend to exhibit lower acceptance and less favourable attitudes toward social robots if they come from a culture with a strong long-term orientation (Papadopoulos et al., 2023). In this study, long-term orientation is defined as the cultural value placed on long-term planning and finding interventions for future improvements, as opposed to focusing on short-term benefits. Thus, further qualitative research is needed to understand cultural needs and how cultural context can shape residents’ attitudes toward technology. Such research is crucial as it allows for the development of more inclusive technology that respects the diverse population that may be exposed to it.

Older adults may perceive the reduction of loneliness through social robot intervention differently compared to another age group (Chen et al., 2024). Therefore, more studies are needed to better understand how older adults perceive loneliness and how technology could accommodate age-specific mental health needs. Importantly, adaptable methods (e.g., conversational interviews) are required to gain older adult perspectives (Wong et al., 2022). Future policies should ensure that social robots are developed and deployed ethically and inclusively. Lastly, evaluation studies on the implementation of social robots should involve residents in LTC to gather their perspectives and address their concerns.

This research has certain limitations. Our work is rooted in a qualitative approach, which included analyzing in-depth case studies, direct prolonged ethnographic observations, and reflective discussions with patients, family partners and experienced clinicians in the context of our research. We acknowledge these ethical challenges represent selected examples drawn from specific cases and contexts. Ethical concerns may vary across cultural contexts, with different types of robots, or in broader investigations involving more LTC homes. Given that these challenges were identified from individual instances, future research should adopt a more systematic approach to data collection. We advocate for pluralism, highlighting that integrating multiple methods, quantitative and qualitative—such as structured and user-led interviews, focus groups, and participatory observation. For a comprehensive, multidimensional understanding of complex ethical phenomena, future research can build on our findings by incorporating additional perspectives through dialogues with LTC professionals, residents, and families, thereby enriching the understanding of ethical issues and enhancing the relevance and applicability of mitigation strategies across diverse contexts. Engaging LTC staff, verbally communicative residents, and family members in discussions on ethical concerns and mitigation strategies could provide a more comprehensive understanding and validation of these issues.

## 5 Conclusion

This paper outlined four ethical challenges (inequitable access, consent, substitution of human care, and concerns about infantilization) and associated mitigation strategies for using social robots in LTC settings. Our findings highlight the importance of adopting an equity and inclusion approach while protecting the residents’ autonomy and dignity. Thus, we advocate for efforts to ensure that social robots are implemented in an ethically inclusive manner to support the mental health and wellbeing of older adults living in LTC.

## Data Availability

The original contributions presented in the study are included in the article/supplementary material, further inquiries can be directed to the corresponding author.

## References

[B1] Banks S. Armstrong A. Carter K. Graham H. Hayward P. Henry A. (2013). Everyday ethics in community-based participatory research. Contemp. Soc. Sci. 8 (3), 263–277. 10.1080/21582041.2013.769618

[B2] Black B. S. Rabins P. V. Sugarman J. Karlawish J. H. (2010). Seeking assent and respecting dissent in dementia research. Am. J. Geriatric Psychiatry 18 (1), 77–85. 10.1097/JGP.0b013e3181bd1de2 PMC2811536 20094021

[B3] Blindheim K. Solberg M. Hameed I. A. Alnes R. E. (2023). Promoting activity in long-term care facilities with the social robot Pepper: a pilot study. Inf. Health Soc. Care 48 (2), 181–195. 10.1080/17538157.2022.2086465 35702818

[B4] Boada J. P. Maestre B. R. Genís C. T. (2021). The ethical issues of social assistive robotics: a critical literature review. Technol. Soc. 67, 101726. 10.1016/j.techsoc.2021.101726

[B5] Chen S.-C. Jones C. Moyle W. (2024). The impact of engagement with the PARO therapeutic robot on the psychological benefits of older adults with dementia. Clin. Gerontol. 47 (5), 909–921. 10.1080/07317115.2022.2117674 36062840

[B6] Chita-Tegmark M. Scheutz M. (2021). Assistive robots for the social management of health: a framework for robot design and human–robot interaction research. Int. J. Soc. Robotics 13 (2), 197–217. 10.1007/s12369-020-00634-z PMC7223628 32421077

[B7] Dewing J. (2007). Participatory research. Dementia 6 (1), 11–25. 10.1177/1471301207075625

[B8] Dinesen B. Hansen H. K. Grønborg G. B. Dyrvig A.-K. Leisted S. D. Stenstrup H. (2022). Use of a social robot (LOVOT) for persons with dementia: exploratory study. JMIR Rehabilitation Assistive Technol. 9 (3), e36505. 10.2196/36505 PMC9379791 35916689

[B9] Fardeau E. Senghor A. S. Racine E. (2023). The impact of socially assistive robots on human flourishing in the context of dementia: a scoping review. Int. J. Soc. Robotics 15 (6), 1025–1075. 10.1007/s12369-023-00980-8 PMC10115607 37359430

[B10] Groove X. (2024). Technology. LOVOT. Tokyo, Japan: Inc. Available online at: https://lovot.life/en/technology/.

[B11] Grunfelder J. Norlén G. Randall L. Gassen N. S. (2020). State of the nordic region 2020. Nordic Council of Ministers. 10.6027/NO2020-001

[B12] Hung L. Berndt A. Wallsworth C. Horne N. Gregorio M. Mann J. (2019a). Involving patients and families in a social robot study. Patient Exp. J. 6 (2), 66–74. 10.35680/2372-0247.1362

[B13] Hung L. Chaudhury H. (2019). It’s my buddy: exploring the perceptions of people with dementia about the social robot PARO in a hospital setting. Innovation Aging 3 (Suppl. ment_1), S331. 10.1093/geroni/igz038.1206 PMC7983329 31822130

[B14] Hung L. Gregorio M. Mann J. Wallsworth C. Horne N. Berndt A. (2021). Exploring the perceptions of people with dementia about the social robot PARO in a hospital setting. Dementia 20 (2), 485–504. 10.1177/1471301219894141 31822130 PMC7983329

[B15] Hung L. Ito H. Wong J. (2023). LOVOT robot as companions for older adults in long-term care. Innovation Aging 7 (Suppl. ment_1), 1077. 10.1093/geroni/igad104.3460

[B16] Hung L. Liu C. Woldum E. Au-Yeung A. Berndt A. Wallsworth C. (2019b). The benefits of and barriers to using a social robot PARO in care settings: a scoping review. BMC Geriatr. 19 (1), 232. 10.1186/s12877-019-1244-6 31443636 PMC6708202

[B17] Hung L. Wong J. Wong K. L. Y. Tan K. C.-K. Lou V. W.-Q. (2025). “It’s always happy to see me”: exploring LOVOT robots as companions for older adults. J. Rehabilitation Assistive Technol. Eng. 12, 20556683251320669. 10.1177/20556683251320669 PMC11811964 39935956

[B18] Körtner T. (2016). Ethical challenges in the use of social service robots for elderly people. Z. Für Gerontol. Und Geriatr. 49 (4), 303–307. 10.1007/s00391-016-1066-5 27220734

[B19] Mann J. Hung L. (2019). Co-research with people living with dementia for change. Action Res. 17 (4), 573–590. 10.1177/1476750318787005

[B20] Mikesell L. (2023). An evolving ethical framework for patient and community-engaged research, 27–39. 10.1007/978-3-031-40379-8_3

[B21] Mononen K. (2019). Embodied care: affective touch as a facilitating resource for interaction between caregivers and residents in a care home for older adults. Linguist. Vanguard 5 (s2). 10.1515/lingvan-2018-0036

[B22] Östlund L. Ernsth Bravell M. Johansson L. (2023). Working in a gray area—healthcare staff experiences of receiving consent when caring for persons with dementia. Dementia 22 (1), 144–160. 10.1177/14713012221137472 36380421

[B23] Papadopoulos I. Wright S. Koulouglioti C. Ali S. Lazzarino R. Martín‐García Á. (2023). Socially assistive robots in health and social care: acceptance and cultural factors. Results from an exploratory international online survey. Jpn. J. Nurs. Sci. 20 (2), e12523. 10.1111/jjns.12523 36732396

[B24] Persson M. Iversen C. Redmalm D. (2024). Making robots matter in dementia care: conceptualising the triadic interaction between caregiver, resident and robot animal. Sociol. Health and Illn. 46 (6), 1192–1211. 10.1111/1467-9566.13786 38733615

[B25] Pyer M. Ward A. (2024). Developing a dementia friendly approach to consent in dementia research. Aging and Ment. Health 28 (2), 294–301. 10.1080/13607863.2023.2264216 37885301

[B26] Rashid N. L. A. Leow Y. Klainin-Yobas P. Itoh S. Wu V. X. (2023). The effectiveness of a therapeutic robot, ‘Paro’, on behavioural and psychological symptoms, medication use, total sleep time and sociability in older adults with dementia: a systematic review and meta-analysis. Int. J. Nurs. Stud. 145, 104530. 10.1016/j.ijnurstu.2023.104530 37348392

[B27] Smith K. A. Stajduhar K. (2024). Using relational ethics to approach equity in palliative care. Palliat. Care Soc. Pract. 18, 26323524241293820. 10.1177/26323524241293820 39525427 PMC11544665

[B28] Takayanagi K. Kirita T. Shibata T. (2014). Comparison of verbal and emotional responses of elderly people with mild/moderate dementia and those with severe dementia in responses to seal robot, PARO. Front. Aging Neurosci. 6, 257. 10.3389/fnagi.2014.00257 25309434 PMC4176084

[B29] Tan C. K. Lou V. W. Q. Cheng C. Y. M. He P. C. Khoo V. E. J. (2024). Improving the social well-being of single older adults using the LOVOT social robot: qualitative phenomenological study. JMIR Hum. Factors 11, e56669. 10.2196/56669 39178408 PMC11380060

[B30] Wangmo T. Lipps M. Kressig R. W. Ienca M. (2019). Ethical concerns with the use of intelligent assistive technology: findings from a qualitative study with professional stakeholders. BMC Med. Ethics 20 (1), 98. 10.1186/s12910-019-0437-z 31856798 PMC6924051

[B31] Wanko Keutchafo E. L. Kerr J. Jarvis M. A. (2020). Evidence of nonverbal communication between nurses and older adults: a scoping review. BMC Nurs. 19 (1), 53. 10.1186/s12912-020-00443-9 32550824 PMC7298765

[B32] Wong K. L. Y. Hung L. Wong J. Park J. Alfares H. Zhao Y. (2024). Adoption of artificial intelligence–enabled robots in long-term care homes by health care providers: scoping review. JMIR Aging 7, e55257. 10.2196/55257 39190455 PMC11387915

[B33] Wong K. L. Y. Smith C. To-Miles F. Dunn S. Gregorio M. Wong L. (2022). Timely Considerations of Using the de Jong Gierveld Loneliness Scale with Older Adults Living in Long-Term Care Homes: A Critical Reflection. J. Long Term Care, 163–172. 10.31389/jltc.141

[B34] Yu C. Sommerlad A. Sakure L. Livingston G. (2022). Socially assistive robots for people with dementia: systematic review and meta-analysis of feasibility, acceptability and the effect on cognition, neuropsychiatric symptoms and quality of life. Ageing Res. Rev. 78, 101633. 10.1016/j.arr.2022.101633 35462001

